# Tris(*N*,*N*′-diisopropyl­benzamidinato)cerium(III)

**DOI:** 10.1107/S1600536810042704

**Published:** 2010-10-30

**Authors:** Peter Dröse, Cristian G. Hrib, Steffen Blaurock, Frank T. Edelmann

**Affiliations:** a Chemisches Institut der Otto-von-Guericke-Universität, Universitätsplatz 2, D-39116 Magdeburg, Germany

## Abstract

The title compound, [Ce(C_13_H_19_N_2_)_3_], was obtained in moderate yield (67%) by treatment of anhydrous cerium trichloride with three equivalents of Li[PhC(N^*i*^Pr)_2_] in tetra­hydro­furan. It is the first homoleptic lanthanide complex of this amidinate ligand. The central Ce^III^ ion is coordinated by three chelating benzamidinate anions in a distorted octa­hedral fashion, with Ce—N distances in the narrow range 2.482 (2)–2.492 (2) Å. The dihedral angles between the phenyl rings and the chelating N—C—N units are in the range 73.3–87.9°, thus preventing conjugation between the two π-systems. The mol­ecule is located on a twofold rotation axis, and one of the phenyl rings is equally disordered over two alternative symmetry-equivalent positions around this axis.

## Related literature

For general background to lanthanide coordination complexes, see: Bailey & Pace (2001 ▶); Edelmann *et al.* (2002 ▶); Edelmann (2009 ▶). Wedler *et al.* (1992 ▶) describe complexes related to the title compound. For applications of homoleptic metal amid­inato complexes, see: Lim *et al.* (2003 ▶); Päiväsaari *et al.* (2005 ▶).
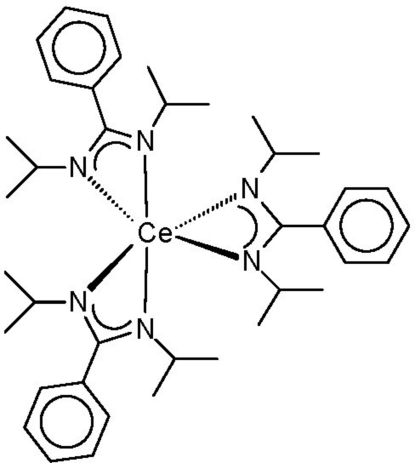


## Experimental

### 

#### Crystal data

[Ce(C_13_H_19_N_2_)_3_]
*M* *_r_* = 750.03
Monoclinic, 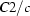

*a* = 14.1225 (4) Å
*b* = 18.5957 (6) Å
*c* = 15.6544 (4) Å
β = 98.324 (2)°
*V* = 4067.8 (2) Å^3^
*Z* = 4
Mo *K*α radiation
μ = 1.15 mm^−1^
*T* = 133 K
0.30 × 0.20 × 0.10 mm

#### Data collection

Stoe IPDS 2T diffractometer
32963 measured reflections
5485 independent reflections
5147 reflections with *I* > 2σ(*I*)
*R*_int_ = 0.048

#### Refinement

*R*[*F* ^2^ > 2σ(*F* ^2^)] = 0.026
*wR*(*F* ^2^) = 0.061
*S* = 1.11
5485 reflections
230 parameters
H-atom parameters constrained
Δρ_max_ = 0.64 e Å^−3^
Δρ_min_ = −1.00 e Å^−3^

### 

Data collection: *X-AREA* (Stoe & Cie, 2002 ▶); cell refinement: *X-AREA*; data reduction: *X-RED* (Stoe & Cie, 2002 ▶); program(s) used to solve structure: *SHELXS97* (Sheldrick, 2008 ▶); program(s) used to refine structure: *SHELXL97* (Sheldrick, 2008 ▶); molecular graphics: *XP* (Siemens, 1994 ▶); software used to prepare material for publication: *SHELXL97*.

## Supplementary Material

Crystal structure: contains datablocks I, global. DOI: 10.1107/S1600536810042704/zl2316sup1.cif

Structure factors: contains datablocks I. DOI: 10.1107/S1600536810042704/zl2316Isup2.hkl

Additional supplementary materials:  crystallographic information; 3D view; checkCIF report

## Figures and Tables

**Fig. 1.**
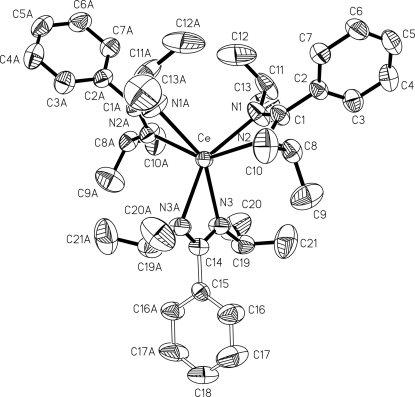
The molecule of the title compound in the crystal. Thermal ellipsoids represent 50% probability levels. The disorder of the phenyl ring C15 - C16A is omitted for clarity.

## supplementary crystallographic information

### Comment

A hot topic in current organolanthanide chemistry is the search for alternative spectator ligands other than cyclopentadienyls which are able to satisfy the coordination requirements of the large lanthanide cations (Edelmann *et al.*, 2002). Among the most successful approaches in this field is the use of amidinate ligands of the general type [RC(NR')_2_]^-^ (*R* = H, alkyl, aryl; *R*' = alkyl, cycloalkyl, aryl, SiMe_3_) which can be regarded as steric cyclopentadienyl equivalents (Bailey & Pace, 2001). Homoleptic lanthanide(III) tris(amidinates) are among the longest known lanthanide complexes containing these chelating ligands. The first lanthanide(III) amidinate complexes ever reported were homoleptic tris(amidinates) of the type [RC_6_H_4_C(NSiMe_3_)_2_]_3_Ln (Wedler *et al.*, 1992). In 2002 it was discovered that homoleptic lanthanide tris(amidinate) complexes display extremely high activity for the ring-opening polymerization of *e*-caprolactone at room temperature. High catalytic activity of homoleptic lanthanide(III) tris(amidinate) has also been found for the polymerization of other polar monomers such as trimethylene carbonate (TMC), lactide, and methylmethacrylate (MMA) (Edelmann, 2009). Even more surprising was the finding that such complexes may turn out to be valuable precursors in materials science and nanotechnology (Edelmann, 2009). For example, pure metals can be deposited by using volatile homoleptic metal amidinato complexes of the type [{MeC(NR')_2_}*_n_*M]*_x_* (*R* = Me, Bu*^t^*; *R* = Pr^i^, Bu*^t^*) and molecular hydrogen gas as the reactants (Lim *et al.*, 2003). Lanthanide amidinate complexes have also been employed in the fabrication of lanthanide-doped inorganic phases by CVD methods (Päiväsaari *et al.*, 2005). We report here the synthesis and structural characterization of a potentially useful homoleptic cerium amidinate, tris[*N,N'*-bis(isopropyl)benzamidinato]cerium(III). Besides X-ray crystallography, the title compound was also characterized by elemental analysis and spectroscopic methods. Due to the paramagnetic nature of the Ce^3+^ ion, the ^1^H NMR signals of the *ortho*-, *meta*- and *para*-phenyl protons appear well separated at δ 12.85, 9.30 and 8.69 p.p.m., respectively, while only broad singlets were observed for the isopropyl protons.

Golden-yellow, highly air-sensitive, block-like single crystals of the title compound were obtained by slow cooling of a saturated solution in *n*-pentane to 278 K. In the solid state the molecule is located on a *C*2 rotation axis, and one of the phenyl rings is disordered over two alternative symmetry equivalent positions around this axis. The coordination geometry around the central cerium(3+) ion can be described as distorted octahedral. The average Ce—N distance is 2.487 Å. With 53.95 (5) - 54.11 (7) the N—Ce—N angles are in the range which is typical for homoleptic lanthanide tris(amidinate) complexes (Edelmann, 2009). The dihedral angles between the phenyl rings and the chelating N—C—N units are in the range of 73.3 - 87.9 °, thus preventing conjugation between the two π -systems.

### Experimental

*Preparation of tris[N,N'-bis(isopropyl)benzamidinato]cerium(III)*: A 100 ml Schlenk-flask was charged with lithium *N,N'*-bis(isopropyl)benzamidinate (3.00 g, 14.3 mmol), anhydrous cerium(III) trichloride (1.17 g, 4.8 mmol) and 100 ml of THF, and the mixture was stirred for 3 h at 333 K, causing the development of a bright yellow color. The mixture was evaporated to dryness and the residue was extracted with *n*-pentane (3 *x* 15 ml) followed by filtration. The clear filtrate was concentrated *in vacuo* to a total volume of *ca* 10 ml. Cooling to 237 K for 24 h afforded 2.42 g (67%) of tris[*N,N'*-bis(isopropyl)benzamidinato]cerium(III) as golden-yellow, block-like crystals. X-ray quality single crystals were grown from a saturated solution in *n*-pentane at 278 K. M. p. 486 K. Anal. calcd for C_39_H_57_CeN_6_ (750.03 g/mol): C 62.45, H 7.66, N 11.20; found: C 62.08, H 7.79, N 10.82%. IR (KBr pellet): *ν*_max_ 3080 (w), 3061 (w), 3022 (w), 2957 (vs, n_as_ CH_3_), 2916 (st), 2888 (st, n_as_ CH_3_), 2861 (st), 1636 (m, NCN unit), 1600 (m, CH ring), 1578 (m, CH ring), 1453 (vs_br_, d_as_ CH_3_), 1374 (vs, NCN unit), 1359 (vs), 1335 (vs), 1274 (w), 1208 (vs), 1166 (st, CH ring), 1133 (vs), 1122 (st), 1073 (w), 1005 (vs), 946 (w), 910 (w), 778 (st), 733 (*m*), 700 (vs, CH ring), 469 (w) cm^-1^. ^1^H NMR (400 MHz, C_6_D_6_, 298 K): δ = 12.85 (d, ^3^*J* = 4.7 Hz, 6H, Ar–*H*), 10.84 (s, 6H, ((CH_3_)_2_C*H*N)_2_CPh), 9.30 (s, 6H, Ar–*H*), 8.69 (t, ^3^*J* = 7.4 Hz, 3H, Ar–*H*), -3.29 (s br, 36H, ((C*H*_3_)_2_CHN)_2_CPh). ^13^C{^1^H} NMR (100.6 MHz, C_6_D_6_, 298 K): δ = 186.9 ((*^i^*PrN)_2_*C*Ph), 148.52 (Ar-*C*), 132.8 (Ar-*C*), 131.6 (Ar-*C*), 130.3 (Ar-*C*), 52.8 ((CH_3_)_2_*C*HN)_2_CPh), 22.7 ((*C*H_3_)_2_CHN)_2_CPh). EI—MS: *m/z* 749.7 (40) [*M*]^+^, 546.3 (100) [*M* – (*^i^*PrN)_2_CPh]^+^, 203.2 (40) [(*^i^*PrN)_2_CPh]^+^, 104.1 (80) [HNCPh]^+^.

### Refinement

One of the phenyl rings is disordered over two alternative symmetry equivalent positions around the two fold rotation axis the molecule is located on. The disordered phenyl ring was contrained to resemble an ideal hexagon with C—C distances of 1.39 Å. No restraints for atom positions or ADPs needed to be applied.

The hydrogen atoms were included using a riding model, with aromatic C—H = 0.95 Å, methyn C—H = 1.00 Å [*U*_iso_(H) = 1.2Ueq(C)] and methyl C—H = 0.98 Å [*U*_iso_(H) = 1.5Ueq(C)].

### Crystal data

**Table:**

[Ce(C_13_H_19_N_2_)_3_]	*F*(000) = 1564
*M**_r_* = 750.03	*D*_x_ = 1.225 Mg m^−^^3^
Monoclinic, *C*2/*c*	Mo *K*α radiation, λ = 0.71073 Å
*a* = 14.1225 (4) Å	Cell parameters from 58857 reflections
*b* = 18.5957 (6) Å	θ = 2.1–29.6°
*c* = 15.6544 (4) Å	µ = 1.15 mm^−^^1^
β = 98.324 (2)°	*T* = 133 K
*V* = 4067.8 (2) Å^3^	Prism, yellow
*Z* = 4	0.30 × 0.20 × 0.10 mm

### Data collection

**Table:**

Stoe IPDS 2T diffractometer	5147 reflections with *I* > 2σ(*I*)
Radiation source: fine-focus sealed tube	*R*_int_ = 0.048
plane graphite	θ_max_ = 29.2°, θ_min_ = 2.1°
Detector resolution: 6.67 pixels mm^-1^	*h* = −19→19
rotation method scans	*k* = −25→25
32963 measured reflections	*l* = −21→20
5485 independent reflections	

### Refinement

**Table:**

Refinement on *F*^2^	Primary atom site location: structure-invariant direct methods
Least-squares matrix: full	Secondary atom site location: difference Fourier map
*R*[*F*^2^ > 2σ(*F*^2^)] = 0.026	Hydrogen site location: inferred from neighbouring sites
*wR*(*F*^2^) = 0.061	H-atom parameters constrained
*S* = 1.11	*w* = 1/[σ^2^(*F*_o_^2^) + (0.031*P*)^2^ + 1.6552*P*] where *P* = (*F*_o_^2^ + 2*F*_c_^2^)/3
5485 reflections	(Δ/σ)_max_ = 0.002
230 parameters	Δρ_max_ = 0.64 e Å^−^^3^
0 restraints	Δρ_min_ = −1.00 e Å^−^^3^

### Special details

**Table:**

Geometry. All e.s.d.'s (except the e.s.d. in the dihedral angle between two l.s. planes) are estimated using the full covariance matrix. The cell e.s.d.'s are taken into account individually in the estimation of e.s.d.'s in distances, angles and torsion angles; correlations between e.s.d.'s in cell parameters are only used when they are defined by crystal symmetry. An approximate (isotropic) treatment of cell e.s.d.'s is used for estimating e.s.d.'s involving l.s. planes.
Refinement. Refinement of *F*^2^ against ALL reflections. The weighted *R*-factor *wR* and goodness of fit *S* are based on *F*^2^, conventional *R*-factors *R* are based on *F*, with *F* set to zero for negative *F*^2^. The threshold expression of *F*^2^ > σ(*F*^2^) is used only for calculating *R*-factors(gt) *etc*. and is not relevant to the choice of reflections for refinement. *R*-factors based on *F*^2^ are statistically about twice as large as those based on *F*, and *R*- factors based on ALL data will be even larger. The phenyl ring with two carbon atoms located on the *C*2 rotation axis lies in two alternative symmetry related positions.

### Fractional atomic coordinates and isotropic or equivalent isotropic displacement parameters (Å^2^)

**Table:**

	*x*	*y*	*z*	*U*_iso_*/*U*_eq_	Occ. (<1)
Ce	0.5000	0.699350 (7)	0.7500	0.03398 (5)	
N1	0.56280 (13)	0.78730 (10)	0.86347 (14)	0.0595 (5)	
N2	0.41661 (11)	0.74104 (9)	0.87036 (11)	0.0465 (4)	
N3	0.57059 (11)	0.57999 (8)	0.79492 (11)	0.0471 (4)	
C1	0.48506 (13)	0.78591 (10)	0.90212 (13)	0.0444 (4)	
C2	0.47606 (12)	0.83394 (10)	0.97814 (12)	0.0413 (4)	
C3	0.52818 (15)	0.82015 (13)	1.05786 (14)	0.0526 (4)	
H3	0.5700	0.7799	1.0648	0.063*	
C4	0.52048 (16)	0.86400 (15)	1.12785 (15)	0.0599 (5)	
H4	0.5566	0.8536	1.1825	0.072*	
C5	0.46115 (17)	0.92218 (13)	1.11859 (15)	0.0592 (5)	
H5	0.4554	0.9521	1.1667	0.071*	
C6	0.40972 (18)	0.93729 (12)	1.03927 (16)	0.0606 (5)	
H6	0.3692	0.9783	1.0325	0.073*	
C7	0.41652 (16)	0.89307 (11)	0.96895 (14)	0.0521 (4)	
H7	0.3801	0.9036	0.9145	0.062*	
C8	0.33639 (13)	0.72355 (12)	0.91592 (13)	0.0475 (4)	
H8	0.3350	0.7586	0.9641	0.057*	
C9	0.3499 (2)	0.64907 (17)	0.9530 (2)	0.0829 (9)	
H9A	0.3575	0.6149	0.9068	0.124*	
H9B	0.2938	0.6357	0.9798	0.124*	
H9C	0.4071	0.6480	0.9966	0.124*	
C10	0.24360 (16)	0.7286 (2)	0.85526 (17)	0.0734 (7)	
H10A	0.2345	0.7780	0.8338	0.110*	
H10B	0.1904	0.7153	0.8860	0.110*	
H10C	0.2454	0.6958	0.8065	0.110*	
C11	0.63744 (17)	0.84142 (15)	0.88303 (17)	0.0688 (7)	
H11	0.6314	0.8656	0.9391	0.083*	
C12	0.6317 (3)	0.89570 (16)	0.8125 (3)	0.1140 (15)	
H12A	0.6431	0.8721	0.7589	0.171*	
H12B	0.6803	0.9329	0.8280	0.171*	
H12C	0.5680	0.9178	0.8040	0.171*	
C13	0.7336 (2)	0.8035 (2)	0.8890 (3)	0.1099 (14)	
H13A	0.7361	0.7641	0.9309	0.165*	
H13B	0.7851	0.8378	0.9076	0.165*	
H13C	0.7414	0.7840	0.8322	0.165*	
C14	0.5000	0.54307 (13)	0.7500	0.0408 (5)	
C15	0.5009 (10)	0.46299 (10)	0.7603 (6)	0.0382 (13)	0.50
C16	0.4568 (8)	0.4312 (3)	0.8245 (4)	0.068 (2)	0.50
H16	0.4272	0.4603	0.8630	0.081*	0.50
C17	0.4559 (5)	0.3567 (3)	0.8326 (3)	0.088 (2)	0.50
H17	0.4258	0.3350	0.8765	0.105*	0.50
C18	0.4992 (6)	0.31412 (11)	0.7763 (4)	0.074 (2)	0.50
H18	0.4986	0.2633	0.7818	0.089*	0.50
C17A	0.5434 (4)	0.3460 (2)	0.7121 (3)	0.0754 (19)	0.50
H17A	0.5729	0.3168	0.6736	0.090*	0.50
C16A	0.5442 (7)	0.4204 (2)	0.7040 (4)	0.0574 (15)	0.50
H16A	0.5744	0.4421	0.6601	0.069*	0.50
C19	0.64510 (15)	0.54478 (12)	0.85451 (16)	0.0561 (5)	
H19	0.6431	0.4920	0.8424	0.067*	
C20	0.74139 (19)	0.5732 (2)	0.8419 (3)	0.0961 (11)	
H20A	0.7531	0.5625	0.7830	0.144*	
H20B	0.7909	0.5502	0.8833	0.144*	
H20C	0.7432	0.6253	0.8511	0.144*	
C21	0.6265 (3)	0.5570 (3)	0.9468 (2)	0.1027 (12)	
H21A	0.6280	0.6086	0.9593	0.154*	
H21B	0.6759	0.5326	0.9869	0.154*	
H21C	0.5635	0.5376	0.9536	0.154*	

### Atomic displacement parameters (Å^2^)

**Table:**

	*U*^11^	*U*^22^	*U*^33^	*U*^12^	*U*^13^	*U*^23^
Ce	0.03793 (7)	0.03059 (7)	0.03552 (7)	0.000	0.01243 (5)	0.000
N1	0.0504 (9)	0.0655 (11)	0.0698 (12)	−0.0243 (8)	0.0333 (8)	−0.0329 (9)
N2	0.0442 (7)	0.0518 (9)	0.0486 (8)	−0.0133 (7)	0.0235 (7)	−0.0136 (7)
N3	0.0468 (8)	0.0351 (7)	0.0554 (9)	0.0003 (6)	−0.0058 (7)	0.0001 (6)
C1	0.0440 (8)	0.0453 (9)	0.0476 (9)	−0.0064 (7)	0.0195 (7)	−0.0107 (7)
C2	0.0411 (8)	0.0422 (9)	0.0439 (9)	−0.0063 (7)	0.0174 (7)	−0.0080 (7)
C3	0.0478 (10)	0.0607 (11)	0.0509 (11)	0.0047 (9)	0.0124 (8)	−0.0057 (9)
C4	0.0549 (11)	0.0809 (15)	0.0447 (10)	−0.0078 (11)	0.0102 (9)	−0.0118 (10)
C5	0.0656 (12)	0.0619 (13)	0.0543 (11)	−0.0135 (10)	0.0229 (10)	−0.0223 (10)
C6	0.0748 (14)	0.0459 (10)	0.0651 (13)	0.0059 (10)	0.0232 (11)	−0.0104 (10)
C7	0.0615 (11)	0.0483 (10)	0.0481 (10)	0.0035 (9)	0.0138 (8)	−0.0037 (8)
C8	0.0432 (9)	0.0557 (10)	0.0478 (10)	−0.0125 (8)	0.0215 (8)	−0.0093 (8)
C9	0.0660 (15)	0.0777 (18)	0.112 (2)	−0.0061 (13)	0.0372 (15)	0.0276 (17)
C10	0.0447 (11)	0.117 (2)	0.0627 (14)	−0.0066 (12)	0.0206 (10)	−0.0024 (15)
C11	0.0597 (12)	0.0783 (16)	0.0766 (15)	−0.0355 (12)	0.0377 (11)	−0.0441 (13)
C12	0.093 (2)	0.0517 (15)	0.193 (4)	−0.0297 (15)	0.006 (3)	0.003 (2)
C13	0.0566 (15)	0.127 (3)	0.139 (4)	−0.0321 (17)	−0.0075 (19)	0.001 (3)
C14	0.0412 (11)	0.0337 (11)	0.0476 (13)	0.000	0.0075 (10)	0.000
C15	0.0347 (11)	0.0388 (12)	0.041 (4)	0.004 (2)	0.005 (3)	−0.0062 (17)
C16	0.079 (4)	0.047 (3)	0.087 (6)	0.006 (3)	0.043 (5)	0.005 (3)
C17	0.097 (5)	0.056 (3)	0.120 (7)	0.005 (3)	0.053 (5)	0.017 (4)
C18	0.076 (3)	0.0347 (19)	0.113 (7)	−0.001 (2)	0.021 (5)	0.004 (2)
C17A	0.089 (4)	0.035 (3)	0.111 (6)	0.008 (3)	0.045 (4)	−0.013 (3)
C16A	0.071 (3)	0.038 (2)	0.070 (4)	0.003 (2)	0.035 (3)	−0.004 (2)
C19	0.0502 (10)	0.0450 (10)	0.0678 (13)	0.0025 (8)	−0.0093 (9)	0.0033 (9)
C20	0.0523 (13)	0.106 (2)	0.124 (3)	−0.0045 (14)	−0.0092 (15)	0.042 (2)
C21	0.098 (2)	0.143 (3)	0.0621 (17)	0.027 (2)	−0.0057 (16)	0.018 (2)

### Geometric parameters (Å, °)

**Table:**

Ce—N1	2.4820 (17)	C10—H10B	0.9800
Ce—N1^i^	2.4820 (17)	C10—H10C	0.9800
Ce—N2	2.4860 (14)	C11—C12	1.490 (5)
Ce—N2^i^	2.4860 (14)	C11—C13	1.521 (5)
Ce—N3^i^	2.4924 (15)	C11—H11	1.0000
Ce—N3	2.4924 (15)	C12—H12A	0.9800
Ce—C14	2.906 (2)	C12—H12B	0.9800
Ce—C1	2.9071 (18)	C12—H12C	0.9800
Ce—C1^i^	2.9071 (18)	C13—H13A	0.9800
N1—C1	1.328 (2)	C13—H13B	0.9800
N1—C11	1.458 (2)	C13—H13C	0.9800
N2—C1	1.319 (2)	C14—N3^i^	1.325 (2)
N2—C8	1.460 (2)	C14—C15^i^	1.498 (3)
N3—C14	1.325 (2)	C14—C15	1.498 (3)
N3—C19	1.457 (2)	C15—C16	1.3900
C1—C2	1.508 (2)	C15—C16A	1.3900
C2—C3	1.378 (3)	C16—C17	1.3900
C2—C7	1.379 (3)	C16—H16	0.9500
C3—C4	1.383 (3)	C17—C18	1.3900
C3—H3	0.9500	C17—H17	0.9500
C4—C5	1.363 (4)	C18—C17A	1.3900
C4—H4	0.9500	C18—H18	0.9500
C5—C6	1.374 (4)	C17A—C16A	1.3900
C5—H5	0.9500	C17A—H17A	0.9500
C6—C7	1.389 (3)	C16A—H16A	0.9500
C6—H6	0.9500	C19—C20	1.498 (4)
C7—H7	0.9500	C19—C21	1.522 (4)
C8—C9	1.503 (4)	C19—H19	1.0000
C8—C10	1.506 (3)	C20—H20A	0.9800
C8—H8	1.0000	C20—H20B	0.9800
C9—H9A	0.9800	C20—H20C	0.9800
C9—H9B	0.9800	C21—H21A	0.9800
C9—H9C	0.9800	C21—H21B	0.9800
C10—H10A	0.9800	C21—H21C	0.9800
			
N1—Ce—N1^i^	97.57 (11)	C8—C9—H9C	109.5
N1—Ce—N2	53.95 (5)	H9A—C9—H9C	109.5
N1^i^—Ce—N2	100.23 (6)	H9B—C9—H9C	109.5
N1—Ce—N2^i^	100.23 (6)	C8—C10—H10A	109.5
N1^i^—Ce—N2^i^	53.95 (5)	C8—C10—H10B	109.5
N2—Ce—N2^i^	143.66 (8)	H10A—C10—H10B	109.5
N1—Ce—N3^i^	151.12 (7)	C8—C10—H10C	109.5
N1^i^—Ce—N3^i^	107.34 (7)	H10A—C10—H10C	109.5
N2—Ce—N3^i^	106.11 (6)	H10B—C10—H10C	109.5
N2^i^—Ce—N3^i^	106.13 (6)	N1—C11—C12	110.6 (2)
N1—Ce—N3	107.34 (7)	N1—C11—C13	107.7 (2)
N1^i^—Ce—N3	151.12 (7)	C12—C11—C13	108.2 (3)
N2—Ce—N3	106.13 (6)	N1—C11—H11	110.1
N2^i^—Ce—N3	106.11 (6)	C12—C11—H11	110.1
N3^i^—Ce—N3	54.12 (7)	C13—C11—H11	110.1
N1—Ce—C14	131.22 (6)	C11—C12—H12A	109.5
N1^i^—Ce—C14	131.22 (6)	C11—C12—H12B	109.5
N2—Ce—C14	108.17 (4)	H12A—C12—H12B	109.5
N2^i^—Ce—C14	108.17 (4)	C11—C12—H12C	109.5
N1^i^—Ce—C1	99.24 (7)	H12A—C12—H12C	109.5
N2^i^—Ce—C1	123.14 (6)	H12B—C12—H12C	109.5
N3^i^—Ce—C1	130.59 (6)	C11—C13—H13A	109.5
N3—Ce—C1	109.60 (6)	C11—C13—H13B	109.5
C14—Ce—C1	123.62 (4)	H13A—C13—H13B	109.5
N1—Ce—C1^i^	99.24 (7)	C11—C13—H13C	109.5
N2—Ce—C1^i^	123.14 (6)	H13A—C13—H13C	109.5
N3^i^—Ce—C1^i^	109.60 (6)	H13B—C13—H13C	109.5
N3—Ce—C1^i^	130.59 (6)	N3—C14—N3^i^	117.6 (2)
C14—Ce—C1^i^	123.62 (4)	N3—C14—C15^i^	124.5 (6)
C1—Ce—C1^i^	112.76 (8)	N3^i^—C14—C15^i^	117.6 (6)
C1—N1—C11	122.56 (17)	N3—C14—C15	117.6 (6)
C1—N1—Ce	94.58 (12)	N3^i^—C14—C15	124.5 (6)
C11—N1—Ce	141.07 (13)	N3—C14—Ce	58.81 (11)
C1—N2—C8	122.34 (16)	N3^i^—C14—Ce	58.81 (11)
C1—N2—Ce	94.66 (10)	C15^i^—C14—Ce	173.9 (3)
C8—N2—Ce	141.91 (12)	C15—C14—Ce	173.9 (3)
C14—N3—C19	121.67 (16)	C16—C15—C16A	120.0
C14—N3—Ce	94.14 (12)	C16—C15—C14	120.2 (5)
C19—N3—Ce	143.45 (13)	C16A—C15—C14	119.8 (5)
N2—C1—N1	116.73 (16)	C17—C16—C15	120.0
N2—C1—C2	122.10 (15)	C17—C16—H16	120.0
N1—C1—C2	121.17 (16)	C15—C16—H16	120.0
N2—C1—Ce	58.46 (9)	C16—C17—C18	120.0
N1—C1—Ce	58.33 (10)	C16—C17—H17	120.0
C2—C1—Ce	177.18 (14)	C18—C17—H17	120.0
C3—C2—C7	118.72 (18)	C17A—C18—C17	120.0
C3—C2—C1	120.63 (18)	C17A—C18—H18	120.0
C7—C2—C1	120.65 (18)	C17—C18—H18	120.0
C2—C3—C4	121.0 (2)	C18—C17A—C16A	120.0
C2—C3—H3	119.5	C18—C17A—H17A	120.0
C4—C3—H3	119.5	C16A—C17A—H17A	120.0
C5—C4—C3	120.1 (2)	C17A—C16A—C15	120.0
C5—C4—H4	120.0	C17A—C16A—H16A	120.0
C3—C4—H4	120.0	C15—C16A—H16A	120.0
C4—C5—C6	119.7 (2)	N3—C19—C20	110.0 (2)
C4—C5—H5	120.2	N3—C19—C21	109.4 (2)
C6—C5—H5	120.2	C20—C19—C21	111.0 (3)
C5—C6—C7	120.5 (2)	N3—C19—H19	108.8
C5—C6—H6	119.8	C20—C19—H19	108.8
C7—C6—H6	119.8	C21—C19—H19	108.8
C2—C7—C6	120.0 (2)	C19—C20—H20A	109.5
C2—C7—H7	120.0	C19—C20—H20B	109.5
C6—C7—H7	120.0	H20A—C20—H20B	109.5
N2—C8—C9	109.27 (18)	C19—C20—H20C	109.5
N2—C8—C10	110.12 (17)	H20A—C20—H20C	109.5
C9—C8—C10	110.5 (2)	H20B—C20—H20C	109.5
N2—C8—H8	109.0	C19—C21—H21A	109.5
C9—C8—H8	109.0	C19—C21—H21B	109.5
C10—C8—H8	109.0	H21A—C21—H21B	109.5
C8—C9—H9A	109.5	C19—C21—H21C	109.5
C8—C9—H9B	109.5	H21A—C21—H21C	109.5
H9A—C9—H9B	109.5	H21B—C21—H21C	109.5
			
N1^i^—Ce—N1—C1	95.52 (15)	C14—Ce—C1—N2	−61.08 (14)
N2—Ce—N1—C1	−1.66 (13)	C1^i^—Ce—C1—N2	118.92 (14)
N2^i^—Ce—N1—C1	150.12 (14)	N1^i^—Ce—C1—N1	−88.55 (18)
N3^i^—Ce—N1—C1	−54.1 (2)	N2—Ce—C1—N1	177.0 (2)
N3—Ce—N1—C1	−99.29 (15)	N2^i^—Ce—C1—N1	−35.84 (17)
C14—Ce—N1—C1	−84.48 (15)	N3^i^—Ce—C1—N1	148.96 (14)
C1^i^—Ce—N1—C1	122.86 (14)	N3—Ce—C1—N1	89.91 (15)
N1^i^—Ce—N1—C11	−67.9 (3)	C14—Ce—C1—N1	115.96 (14)
N2—Ce—N1—C11	−165.1 (3)	C1^i^—Ce—C1—N1	−64.04 (14)
N2^i^—Ce—N1—C11	−13.3 (3)	N2—C1—C2—C3	107.5 (2)
N3^i^—Ce—N1—C11	142.4 (3)	N1—C1—C2—C3	−73.0 (3)
N3—Ce—N1—C11	97.3 (3)	N2—C1—C2—C7	−73.4 (3)
C14—Ce—N1—C11	112.1 (3)	N1—C1—C2—C7	106.2 (2)
C1—Ce—N1—C11	−163.4 (4)	C7—C2—C3—C4	0.7 (3)
C1^i^—Ce—N1—C11	−40.5 (3)	C1—C2—C3—C4	179.88 (19)
N1—Ce—N2—C1	1.67 (13)	C2—C3—C4—C5	−0.4 (4)
N1^i^—Ce—N2—C1	−90.29 (14)	C3—C4—C5—C6	−0.5 (4)
N2^i^—Ce—N2—C1	−50.10 (12)	C4—C5—C6—C7	1.1 (4)
N3^i^—Ce—N2—C1	158.16 (12)	C3—C2—C7—C6	−0.2 (3)
N3—Ce—N2—C1	101.64 (13)	C1—C2—C7—C6	−179.3 (2)
C14—Ce—N2—C1	129.90 (12)	C5—C6—C7—C2	−0.8 (4)
C1^i^—Ce—N2—C1	−74.56 (17)	C1—N2—C8—C9	−105.7 (3)
N1—Ce—N2—C8	−165.3 (3)	Ce—N2—C8—C9	58.8 (3)
N1^i^—Ce—N2—C8	102.8 (2)	C1—N2—C8—C10	132.7 (2)
N2^i^—Ce—N2—C8	143.0 (2)	Ce—N2—C8—C10	−62.8 (3)
N3^i^—Ce—N2—C8	−8.8 (2)	C1—N1—C11—C12	−104.8 (3)
N3—Ce—N2—C8	−65.3 (2)	Ce—N1—C11—C12	55.4 (4)
C14—Ce—N2—C8	−37.0 (2)	C1—N1—C11—C13	137.2 (3)
C1—Ce—N2—C8	−166.9 (3)	Ce—N1—C11—C13	−62.6 (4)
C1^i^—Ce—N2—C8	118.5 (2)	C19—N3—C14—N3^i^	172.3 (2)
N1—Ce—N3—C14	155.00 (8)	C19—N3—C14—C15^i^	−13.8 (4)
N1^i^—Ce—N3—C14	−56.63 (17)	Ce—N3—C14—C15^i^	173.9 (3)
N2—Ce—N3—C14	98.47 (9)	C19—N3—C14—C15	−2.0 (4)
N2^i^—Ce—N3—C14	−98.51 (9)	Ce—N3—C14—C15	−174.3 (3)
C1—Ce—N3—C14	126.50 (9)	C19—N3—C14—Ce	172.3 (2)
C1^i^—Ce—N3—C14	−85.72 (11)	N1—Ce—C14—N3	−32.43 (11)
N1—Ce—N3—C19	−14.0 (3)	N1^i^—Ce—C14—N3	147.57 (11)
N1^i^—Ce—N3—C19	134.4 (2)	N2—Ce—C14—N3	−90.02 (10)
N2—Ce—N3—C19	−70.5 (3)	N2^i^—Ce—C14—N3	89.98 (10)
N2^i^—Ce—N3—C19	92.5 (3)	C1—Ce—C14—N3	−65.41 (11)
N3^i^—Ce—N3—C19	−169.0 (3)	C1^i^—Ce—C14—N3	114.59 (11)
C14—Ce—N3—C19	−169.0 (3)	N1—Ce—C14—N3^i^	147.57 (11)
C1—Ce—N3—C19	−42.5 (3)	N1^i^—Ce—C14—N3^i^	−32.43 (11)
C1^i^—Ce—N3—C19	105.3 (3)	N2—Ce—C14—N3^i^	89.98 (10)
C8—N2—C1—N1	167.7 (2)	N2^i^—Ce—C14—N3^i^	−90.02 (10)
Ce—N2—C1—N1	−2.8 (2)	C1—Ce—C14—N3^i^	114.59 (11)
C8—N2—C1—C2	−12.8 (3)	C1^i^—Ce—C14—N3^i^	−65.41 (11)
Ce—N2—C1—C2	176.75 (17)	N3—C14—C15—C16	88.9 (6)
C8—N2—C1—Ce	170.5 (2)	N3^i^—C14—C15—C16	−85.0 (5)
C11—N1—C1—N2	170.5 (2)	C15^i^—C14—C15—C16	−144 (5)
Ce—N1—C1—N2	2.8 (2)	N3—C14—C15—C16A	−92.6 (5)
C11—N1—C1—C2	−9.0 (4)	N3^i^—C14—C15—C16A	93.5 (6)
Ce—N1—C1—C2	−176.75 (17)	C15^i^—C14—C15—C16A	34 (4)
C11—N1—C1—Ce	167.7 (3)	C14—C15—C16—C17	178.5 (11)
N1—Ce—C1—N2	−177.0 (2)	C14—C15—C16A—C17A	−178.5 (11)
N1^i^—Ce—C1—N2	94.41 (13)	C14—N3—C19—C20	134.4 (2)
N2^i^—Ce—C1—N2	147.12 (11)	Ce—N3—C19—C20	−58.5 (4)
N3^i^—Ce—C1—N2	−28.07 (16)	C14—N3—C19—C21	−103.4 (3)
N3—Ce—C1—N2	−87.12 (13)	Ce—N3—C19—C21	63.7 (3)

Symmetry codes: (i) −*x*+1, *y*, −*z*+3/2.

## References

[bb1] Bailey, P. J. & Pace, S. (2001). *Coord. Chem. Rev.* **214**, 91–141.

[bb2] Edelmann, F. T. (2009). *Chem. Soc. Rev.* **38**, 2253–2268. 10.1039/b800100f 19623348

[bb3] Edelmann, F. T., Freckmann, D. M. M. & Schumann, H. (2002). *Chem. Rev.* **102**, 1851–1896. 10.1021/cr010315c 12059256

[bb4] Lim, B. S., Rahtu, A., Park, J.-S. & Gordon, R. G. (2003). *Inorg. Chem.* **42**, 7951–7958. 10.1021/ic0345424 14632513

[bb5] Päiväsaari, J., Dezelah, C. L., Back, D., El-Kaderi, H. M., Heeg, M. J., Putkonen, M., Niinistö, L. & Winter, C. H. (2005). *J. Mater. Chem.* **15**, 4224–4233.

[bb6] Sheldrick, G. M. (2008). *Acta Cryst.* A**64**, 112–122. 10.1107/S0108767307043930 18156677

[bb7] Siemens (1994). *XP* Siemens Analytical X-ray Instruments Inc., Madison, Wisconsin, USA.

[bb8] Stoe & Cie (2002). *X-AREA* and *X-RED* Stoe & Cie, Darmstadt, Germany.

[bb9] Wedler, M., Knösel, F., Pieper, U., Stalke, D., Edelmann, F. T. & Amberger, H.-D. (1992). *Chem. Ber.* **125**, 2171–2181.

